# A Comparative Analysis of Different Finishing and Polishing Devices on Nanofilled, Microfilled, and Hybrid Composite: A Scanning Electron Microscopy and Profilometric Study

**DOI:** 10.5005/jp-journals-10005-1364

**Published:** 2016-09-27

**Authors:** Rishi D Yadav, Deepak Raisingani, Divya Jindal, Rachit Mathur

**Affiliations:** 1 Reader, Department of Conservative Dentistry and Endodontics, New Horizon Dental College and Research Institute, Bilaspur Chhattisgarh, India; 2 Professor and Head, Department of Conservative Dentistry and Endodontics Mahatma Gandhi Dental College and Hospital, Jaipur, Rajasthan India; 3 Postgraduate Student, Department of Conservative Dentistry and Endodontics Mahatma Gandhi Dental College and Hospital, Jaipur, Rajasthan India; 4 Senior Lecturer, Department of Conservative Dentistry and Endodontics Mahatma Gandhi Dental College and Hospital, Jaipur, Rajasthan India

**Keywords:** Ceram X, Esthet-X, Filtek Z250, Surface roughness.

## Abstract

The continuous development of esthetically acceptable adhesive restorative material has made a variety of tooth-colored materials available for clinical use. The advent of visible light polymerizing resin and the use of finer filler particles permit resin composites to be polished to higher degree. The effect of polishing systems on surface finish has been reported to be material-dependent, and the effectiveness of these systems was mostly product-dependent. Hence, the purpose of this study was to evaluate the efficiency of finishing and polishing systems on the surface roughness of nanofilled, microfilled, and hybrid composite restorative materials available in the market.

**How to cite this article:**

Yadav RD, Raisingani D, Jindal D, Mathur R. A Comparative Analysis of Different Finishing and Polishing Devices on Nanofilled, Microfilled, and Hybrid Composite: A Scanning Electron Microscopy and Profilometric Study. Int J Clin Pediatr Dent 2016;9(3):201-208.

## INTRODUCTION

The demand for esthetic restorations has increased substantially in recent years. The continuous development of esthetically acceptable adhesive restorative material has made a variety of tooth-colored materials available for clinical use.

Resin composite materials are available with a variety of filler types that affect their handling characteristics and physical properties.^1^ These resin materials have progressed from macrofills to microfills and from hybrids to microhybrids, and newer materials, such as nanofilled and nanoceramic composite been subsequently introduced into the dental market. “Nanofilled composites” are new type of composite resins that have been produced with nanofiller technology and formulated with nanomer and nanocluster filler particles. Scientific data indicate that nanofilled resin composites lead to higher surface quality and superior polish retention; they also exhibit low wear rate and increased wear resistance, low shrinkage, and high strength. Nanofilled resin composites also possess favorable mechanical properties.^2^

A smooth surface has always been the prime objective of composite restorations not only for esthetic consideration but also for oral health. Threshold surface roughness for bacterial retention is 0.2 μm, below which no further reduction in bacterial accumulation could be expected.^3^ An increase in surface roughness above this threshold roughness however resulted in a simultaneous increase in plaque accumulation, abrasivity and wear kinetics as well as tactile perception, thereby increasing the risk of both caries and periodontal inflammation. Surface roughness influences resistance to staining and the natural gloss of the restoration.

Finishing is the gross contouring of a restoration to obtain desired anatomy, while polishing refers to reduction of roughness and removal of scratches created by the finishing instrument.^4^ The smoothest surface for composite restorations is achieved when using a Mylar strip (Ra = 0) in contact with the restoration during curing.^5^ A variety of instruments are commonly used for finishing and polishing tooth-colored restorative materials, including carbide finishing burs, diamond finishing burs, abrasive impregnated rubber cups and points, aluminum oxide-coated abrasive disks, abrasive strips, and polishing pastes. Each of these instruments or devices leaves the surface of various restorative materials with varying degrees of surface roughness.^2^

In recent years, efforts have been made to analyze the suitability of numerous systems available for the finishing and polishing of various composites. The effect of polishing systems on surface finish has been reported to be material-dependent, and the effectiveness of these systems was mostly product-dependent. Hence, the purpose of this study was to evaluate the efficiency of finishing and polishing systems on the surface roughness of nanofilled, microfilled, and hybrid composite restorative materials available in the market.

## MATERIALS AND METHODS

The tooth-colored restorative materials selected for this study includes three composite materials: Ceram X (CX), Esthet-X (EX) and Filtek Z250 (FZ). Fifty specimens of each composite material were fabricated in the rectangular recesses (5 mm wide × 15 mm long × 2 mm deep) of a customized brass mold and covered with Mylar strips (totally 90 specimens).

A glass slide was placed on the molds and the pressure was applied to extrude the excess material. The composite materials were then polymerized using light curing unit, which was held at a distance of 1 cm from the specimens. The specimens were cured for 40 seconds.

The surface of all the specimens was gross finished with fine finishing diamond bur of 50 μm for 20 seconds, with slow-speed handpiece at 15,000 rpm to obtain standardized surfaces.

*Group I:* Super Snap Rainbow Technique Kit (Shofu) Finishing and Polishing System (Subgroups A, B, C)

All specimens in this group were subjected to Shofu polishing system. Disks in this kit are attached by a metal hub to the autoclavable metal mandrel. The specimens were subjected to finishing disks (violet), polishing disks (green), and finally to super polishing disks (red).

*Group II:* Sof-Lex Pop-on Contouring and Polishing System (Subgroups A, B, C)

All specimens in this group were subjected to Sof-Lex polishing system. Disks in this kit are attached by a metal hub to the autoclavable metal mandrel. The specimens were subjected to medium, fine, and then super fine disk for polishing of the composite block.

*Group III:* Enhance Finishing and Polishing System (Subgroups A, B, C)

All specimens in this group were subjected to Enhance polishing system. For the immediate finishing, the pointed shape was selected. Enhance point was inserted into conventional speed contra-angled handpiece and the finishing was controlled by the pressure applied to the surface of the composite block. This was followed by foam polishing cup used along with Prisma Gloss polishing paste for 30 seconds. A foam polishing cup was then used with Prisma Gloss Extra fine polishing paste again for 30 seconds.

The gross reduction of the excess was done by using diamond carbide burs in all the groups.

### Profiling Procedure

All the specimens were subjected to profiling procedure for measuring the average surface roughness values using a mechanical digital Profilometer (Taylor Hobson Ltd). This device essentially consists of a stylus attached to a long lever arm, which is traced along the surface and records the up and down movement of the stylus. It also allows the quantification of the surface roughness by calculating average surface roughness (Ra) values, which is the arithmetic average height of the roughness component irregularities from the mean line measured within the sampling length; the higher this value, the rougher the surface. Two profilometric measurements were accomplished on each specimen and then averaged to obtain the surface roughness of that specimen.

### Scanning Electron Microscopy (SEM) Evaluation

All the specimens were subjected for SEM evaluation. One specimen of each subgroup was prepared for the SEM (ZIESS EVO 50) evaluation. The specimens were coated with silver in a vacuum evaporator. Photographs of representative areas of the polished surface were taken at 5000× magnifications.

The results were analyzed by calculating the mean and standard deviations for each group. The data of each material were subjected to analysis of variance (ANOVA) followed by Tukey’s high significant difference (HSD) test and Student’s t-test at a p-value of 0.05.

## RESULTS

The surface roughness was measured by using mechanical digital Profilometer.

## STATISTICAL ANALYSIS

### Formula Used

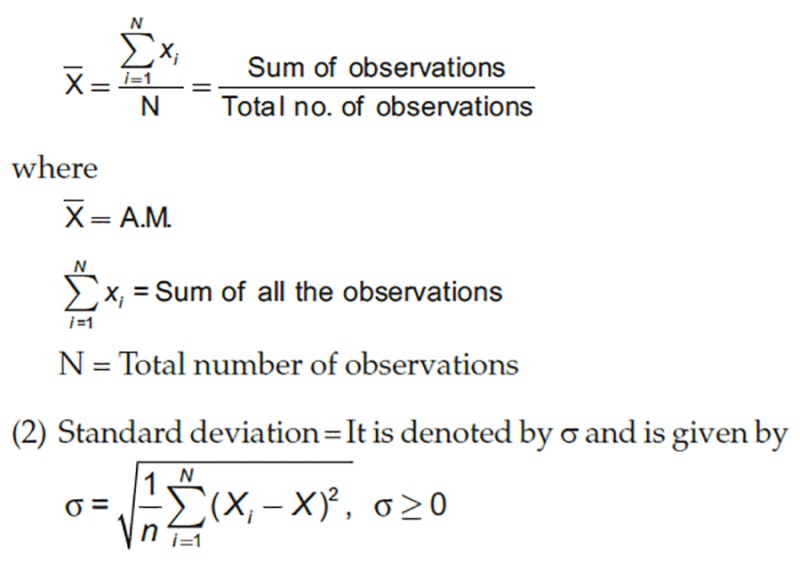

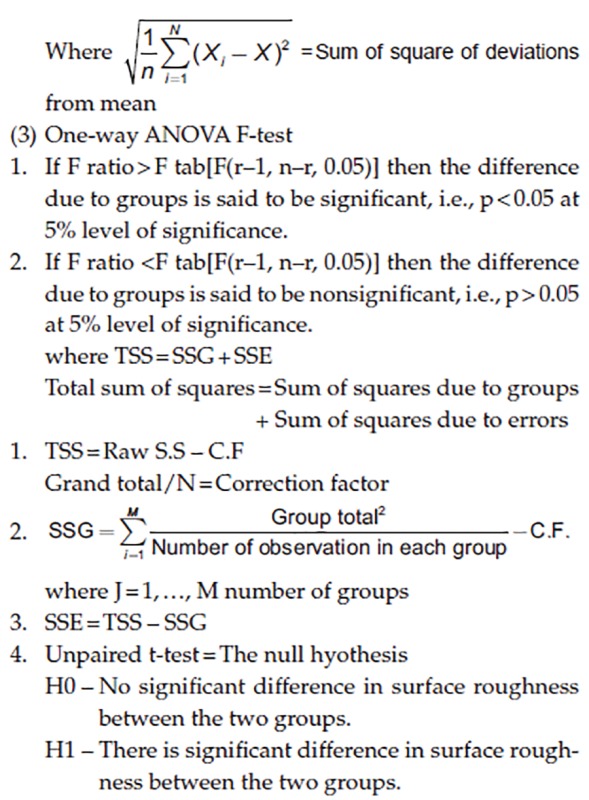

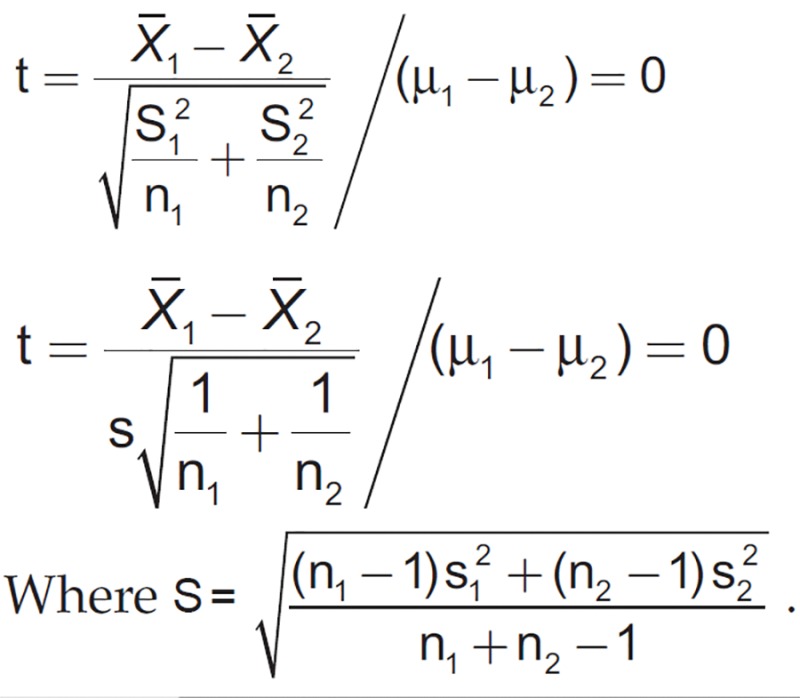







**Table 1A:** **Table 1A:** Comparison of mean surface roughness and standard deviation when composite resins subjected to Sof-Lex polishing system

*Subgroups*		*N*		*Mean*		*S.D.*		*Std. error*		*Min value*		*Max value*	
IIA		10		0.04303		0.005106		0.001615		0.0321		0.0501	
IIB		10		0.06847		0.00351		0.0011		0.0625		0.0729	
IIC		10		0.11254		0.03039		0.009613		0.0887		0.1889	

**Table 1B:** **Table 1B:** Analysis of variance to determine statistical significance of difference in mean for Sof-Lex polishing system

*Square of variation*		*D.F.*		*Sum of* * square (SS)*		*Mean sum of* * square (MSS)*		*F-ratio*		*F-table (2,27,0.05)*		*p-value*	
Between group		2		0.0248		0.0124		41.33		3.37		< 0.05	
Within group		27		0.0087		0.0003							
Total		29		0.03351									

p < 0.05 - significant difference

**Table 2A:** **Table 2A:** Comparison of mean surface roughness and standard deviation when composite resins subjected to Enhance polishing system

*Subgroups*		*N*		*Mean*		*S.D.*		*Std. error*		*Min value*		*Max value*	
IIIA		10		0.1457		0.001863		0.00059		0.1428		0.1491	
IIIB		10		0.5419		0.00115		0.000364		0.5402		0.5438	
IIIC		10		0.2446		0.00113 2		0.000358		0.2428		0.2459	

**Table 2B:** **Table 2B:** Analysis of variance to determine statistical significance of difference in mean for Enhance polishing system

*Square of variation*		*D.F.*		*Sum of* * square (SS)*		*Mean sum of* * square (MSS)*		*F-ratio*		*F-table (2,27,0.05)*		*p-value*	
Between group		2		0.8505		0.4253		141.8		3.37		< 0.05	
Within group		27		0.010		0.00003							
Total		29		0.8515									

p < 0.05 - significant difference

The data obtained in this study were subjected to statistical analysis using one-way ANOVA and Tukey’s HSD test.

One-way ANOVA showed the statistically significant difference in groups II and III respectively, i.e., p < 0.05 (Tables 1A, B and 2A, B). However, no significant difference was observed in group I (Tables 3A and B). Further, no significant difference was observed between the groups while taking simultaneous comparison, i.e., p < 0.05 (Table 4).

According to Tukey’s HSD test for inter subgroup comparison, shofu polishing system showed superior polishability than other polishing system. There was no statistically significant difference in surface roughness obtained by Shofu polishing system. When Tukey’s HSD test was done for inter subgroup comparison within each group of composite resin materials polished, there was no statistically significant difference in polishability obtained by CX composite resin (Tables 1B, 2B, 3B, and 5).

**Table 3A:** **Table 3A:** Comparison of mean surface roughness and standard deviation when composite resins subjected to Shofu polishing system

Subgroups		*N*		*Mean*		*S.D.*		*Std. error*		*Min value*		*Max value*	
IA		10		0.0799		0.005664		0.001792		0.0710		0.0892	
IB		10		0.1361		0.002148		0.0006791		0.1321		0.1395	
IC		10		0.1970		0.001606		0.0005079		0.1952		0.1995	

**Table 3B:** **Table 3B:** Analysis of variance to determine statistical significance of difference in mean for Shofu polishing system

*Square of variation*		*D.F.*		*Sum of* *square (SS)*		*Mean sum of* * square (MSS)*		*F-ratio*		*F-table (2,27,0.05)*		*p-value*	
Between group		2		0.685		0.0343		1.26		3.37		> 0.05	
Within group		27		0.7355		0.0272							
Total		29		0.8040									

p > 0.05 - no significant difference

**Table 4:** **Table 4:** Analysis of variance to determine statistical significance of difference in mean for all the groups

*Square of variation*		*D.F.*		*Sum of* *square (SS)*		*Mean sum of* * square (MSS)*		*F-ratio*		*F-table (4,145,0.05)*		*p-value*	
Between group		2		1.0712		0.2678		36.19		2.37		< 0.05	
Within group		87		1.0688		0.0074							
Total		89		2.1400									

**Table 5:** **Table 5:** Intergroup comparison done using Tukey’s HSD between the groups and subgroups

*Groups*		*Subgroups*		*Observations*		*Mean ± S.D.*		*Groups Mean ±S.D.*		*Std. error*	
I (Shofu)		Nanofilled-IA		10		0.0799 ± 0.005664		0.1377 ± 0.009418		0.0017	
		Microfilled-IB		10		0.1361 ± 0.002148					
		Hybrid-IC		10		0.1970 ± 0.001606					
II (Sof-Lex)		Nanofilled-IIA		10		0.04303 ± 0.005106		0.0747 ± 0.03901		0.0071	
		Microfilled-IIB		10		0.06847 ± 0.00351					
		Hybrid-IIC		10		0.11254 ± 0.03039					
III (Enhance)		Nanofilled-IIIA		10		0.1457 ± 0.001863		0.3108 ± 0.004146		0.00076	
		Microfilled-IIIB		10		0.5419 ± 0.0015					
		Hybrid-IIIC		10		0.2446 ± 0.001132					

## DISCUSSION

The surface micromorphology of resin composites after finishing and polishing has been shown to be influenced by the size, hardness, and amount of filler particles. Harder filler particles are left protruding from the surface during polishing, as the softer resin matrix is preferentially removed in hybrid composites. Filler particles should be situated as close together as possible in order to protect the resin matrix from abrasives. Hence, the application of nanotechnology for the development of newer resin has great potential. Reduced dimension of the particles with wider distribution can achieve increased filler loading, which results in reduced polymerization shrinkage and improved mechanical properties.^4^

The complex structure of a surface cannot be fully characterized by the use of only surface roughness measurement. More valid predictions of clinical performance can be made when the surface roughness measurements are combined with a SEM analysis that permits an evaluation on the destructive potential of a finishing and polishing system.^4^ The SEM uses a focused electron beam which is scanned on the surface of the sample to produce high-quality image of the surface topography. Scanning electron microscopy essentially offers a very high magnification with very high-resolution capabilities and a large depth of focus. This characteristic makes it an indispensable tool for analysis of a wide class of conducting, semi-conducting, and insulating materials.

Photographs of representative areas of the polished surfaces were taken at 5000× magnifications.^4^

The specimens were finished after 24 hours in this study.^6,7^ The final finishing should always be delayed for at least 24 hours when composites are used. If finishing is conducted immediately after composite placement, the material might be more readily subjected to plastic deformation due to the heat generated during the finishing/polishing procedure. Approximately 75% of light-polymerization occurs during the first 10 minutes. The polymerization reaction continues for a 24-hour period if the restoration is immersed in water before finishing procedures.

Among all the composite materials tested, CX (nanofilled composite) showed superior polishability than the Esthet X (EX) (microfilled composite) and Filtek Z250 (hybrid composite) composite materials for all the polishing systems. The Filtek Z250 showed the least polish-ability compared with EX and CX composite restorative materials.

Ceram X composite showed superior polishability probably because the combination of nanomer-sized particles and nanocluster formulations reduces the interstitial spacing of the filler particles and therefore, provides increased filler loading, better physical properties, and improved polish retention.

A nanocluster filler particle consists of loosely bound agglomerates of nano-sized filler particles. During polishing, only the nano-sized filler particles worn away whereas nanocluster are not plucked out from the resin matrix. Eventually, the surface has smaller defects and better polish retention.^2^

Filtek Z250 showed the least surface smoothness compared with Esthet-X and CX composite restorative materials, probably because it contains large glass filler particles which can be plucked away, leaving voids or rougher surface after being polished.^4,7^

Esthet-X showed more surface roughness than CX composite. Esthet-X showed surface roughness, probably because the resin components differ in the incorporation of BisEMA. BisEMA has a high molecular weight and fewer double-bonds resulting in a slightly softer matrix.^5^

According to a study conducted to measure the surface roughness of different types of flowable restorative resins in producing smooth surfaces, there is no differences in surface roughness among the materials tested, except for Dyract Flow, a flowable composite.^5^ The cups and points of Sof-Lex polishing system are unable to flatten the glass filler particles, thus providing rougher surfaces compared with other finishing and polishing systems. Another reason for high surface roughness could be related to the resin components, which differ in the incorporation of BisEMA. BisEMA has a high molecular weight and fewer double-bonds resulting in a slightly softer matrix.

Esthet-X showed better surface smoothness than Filtek Z250, probably because they have less inorganic content with a smaller filler particles than the Filtek Z250 containing larger filler particles and they can be finished and polished to a very smooth surface due to their small filler particle and arrangement.^2,8^ Ceram X showed better pol-ishability, followed by Esthet-X, and Filtek Z250 formula composite respectively.

Recently, diamond polishers and silicon synthetic rubbers have been introduced, which give shine and reduce the clinical time spent to finish the restoration. Manufacturers refer to them as “one-step” polishing system, because they can be used to develop a high luster, and contouring finishing and polishing procedures could be completed using a single instrument.^4^

The coarse disks supplied with Shofu and Sof-Lex Pop-on system are primarily for contouring and gross finishing. Since the aim of our study was finishing and fine finishing (or polishing), the coarse disks were not used. The composite blocks received a standard finish using diamond finishing bur (50 μm). The coarse disks have been commonly used for gross finishing of conventional composite materials which contain larger filler particle size. But in our study, we have used nanofilled composite where the filler particles are smaller than the coated abrasive particle itself. There is no coarse finishing instrument in the Enhance polishing kit; hence, this procedure allowed gross finishing of the specimens.

The polishing systems used in the study are Super Snap Rainbow Technique Kit (Shofu), Sof-Lex Pop-on disks, and Enhance polishing systems. The Shofu polishing system contains finishing disk (Violet), polishing disk (Green), and super polishing disk (Red). The Sof-Lex polishing system contains medium, fine and super fine aluminum disk. The Enhance finishing and polishing system contains finishing points and cups, which contain same particle size (40 μm), and are used according to accessibility in the anterior and posterior regions of the oral cavity.

Equivalent time period of 30 seconds each was used for sequence of three instruments of polishing systems, which was in accordance with the study done by Setcos et al.^9^

According to our study, the smoothest surface is produced by Shofu polishing system, followed by Sof-Lex and Enhance polishing system. Shofu polishing disks produced smoother surface, probably because the aluminum oxide disks appear to finish or tend to sand the surface material without dislodging the glass particles. Malleability of Shofu aluminum oxide disks also promotes a homogeneous abrasion of filler and resin matrix.^9^

The Enhance polishing system showed the least pol-ishability among all the polishing systems used, probably because it abrade softer resin matrices at a higher rate and harder filler particles are left protruding from the surfaces. The cups and points are unable to flatten the glass filler particles, thus providing rougher surfaces compared with other finishing and polishing systems. The polishing cups in the Enhance polishing system seemed to cause displacement of filler particles and also grind into the surface causing rougher surface.^2,10^

The Sof-Lex Pop-on disks showed smoother surface than Enhance polishing system.^8,11,12^ Large particles embedded in Sof-Lex disks tend to rip through the surface of the composites and when used with certain hybrid composites tend to cut and abrade filler particles and resin matrix equally, resulting in smoother surface. But the main disadvantage is that the frictional heat generated by Sof-Lex disks causes micro cracks in polymer matrix, which gives rougher surface for hybrid composite materials.^13^

**Fig. 1:**
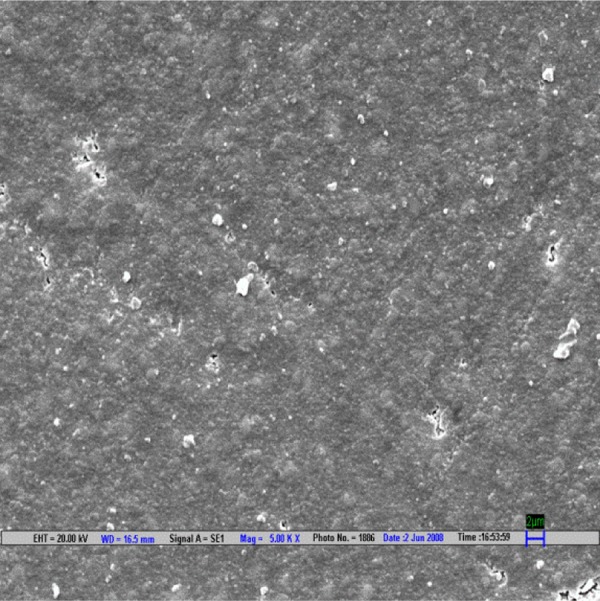
Scanning electron microscopic image of CX composite polished by Shofu polishing system (magnification 5000*)

**Fig. 2:**
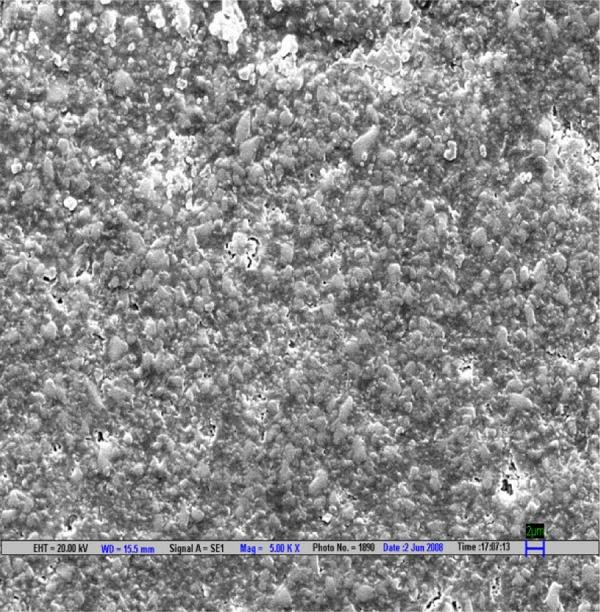
Scanning electron microscopic image of EX composite polished by Shofu polishing system (magnification 5000*)

**Fig. 3:**
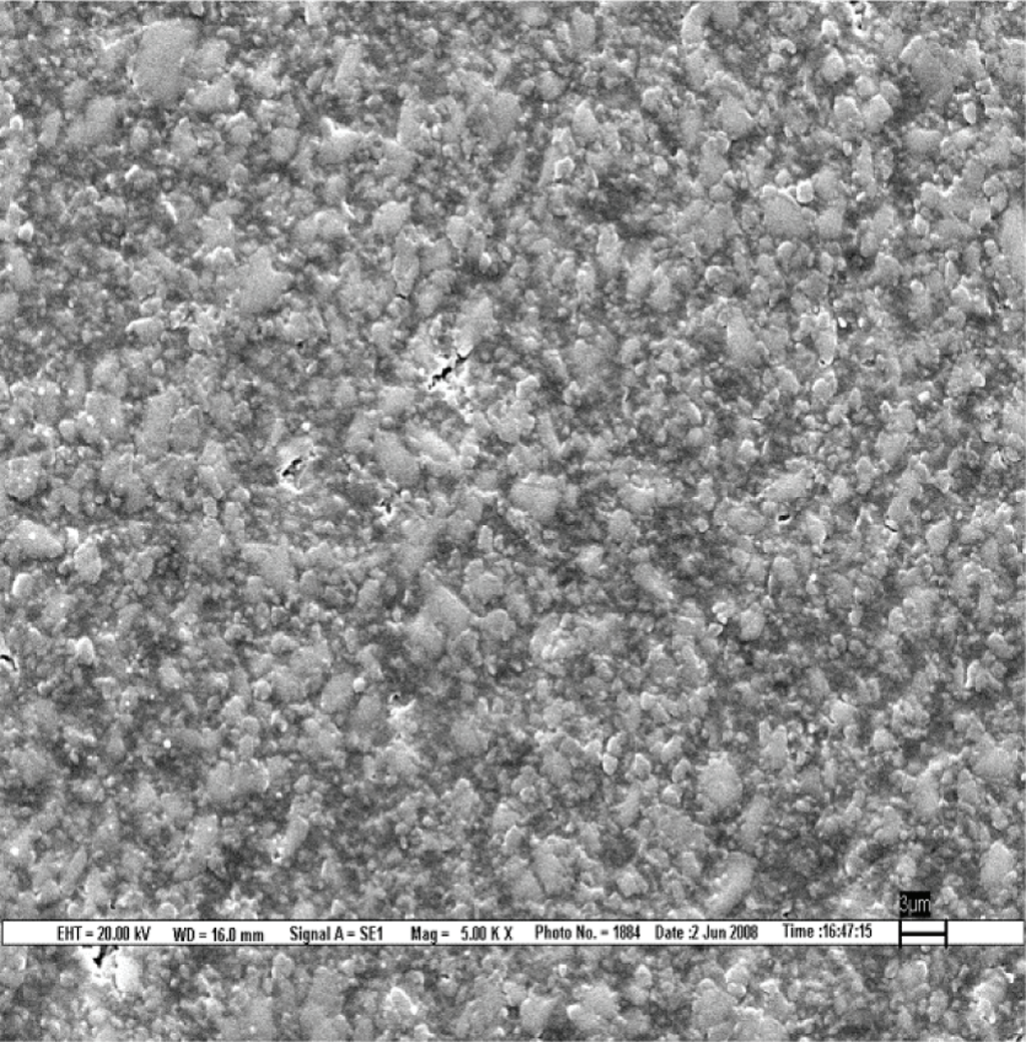
Scanning electron microscopic image of Filtek Z 250 composite polished by Shofu polishing system (magnification 5000*)

**Fig. 4:**
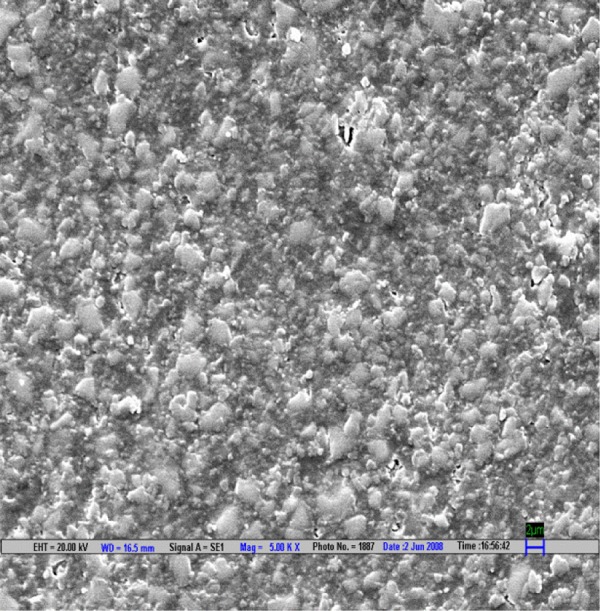
Scanning electron microscopic image of CX composite polished by Sof-Lex polishing system (magnification 5000*)

The result is evaluated by SEM at 5000× (Figs 1 to 9).

Scanning electron microscopy pictures of the polished surfaces shows less scratches and pitting for the group I (Shofu) and CX composite when compared with other polishing systems.^4^

According to the SEM images, the Enhance polishing system and hybrid composite (Filtek Z-250) show more scratches and pitting because large glass fillers were plucked away, leaving voids or craters behind after being polished.^4^

**Fig. 5:**
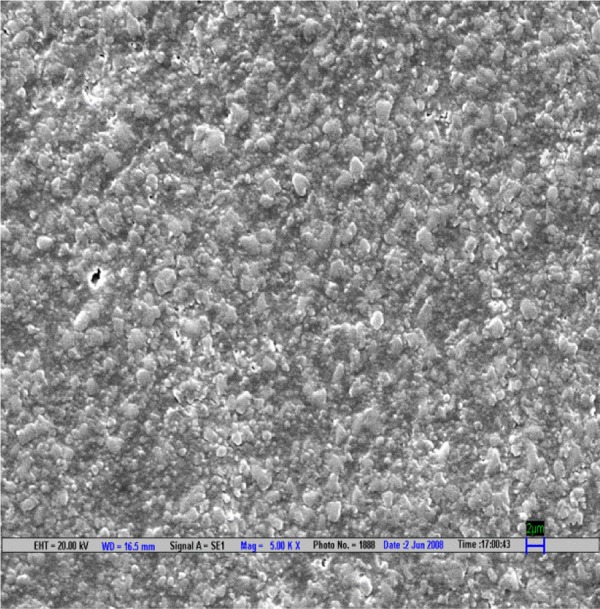
Scanning electron microscopic image of EX composite polished by Sof-Lex polishing system (magnification 5000*)

**Fig. 6:**
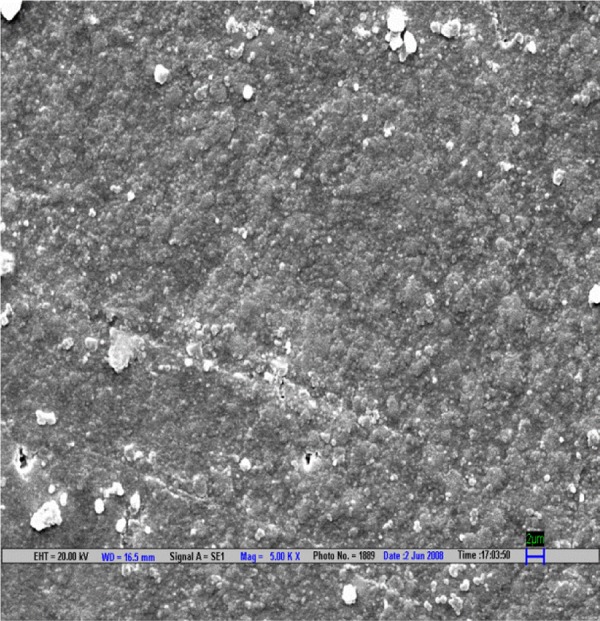
Scanning electron microscopic image of Filtek Z 250 composite polished by Sof-Lex polishing system (magnification 5000*)

**Fig. 7:**
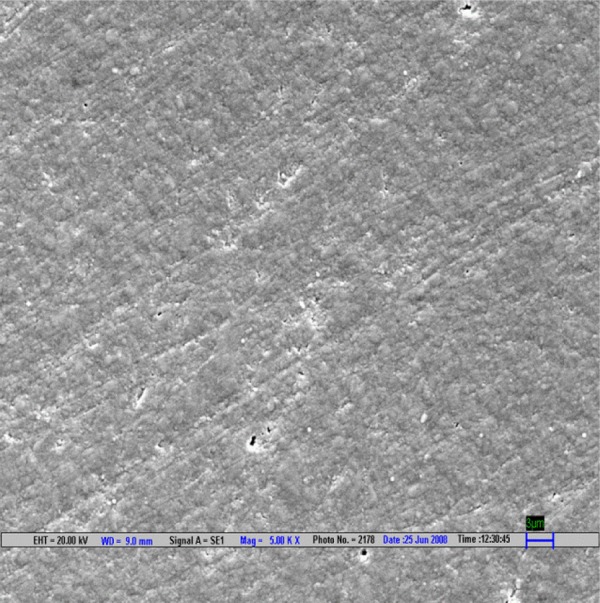
Scanning electron microscopic image of CX composite polished by Enhance polishing system (magnification 5000*)

**Fig. 8:**
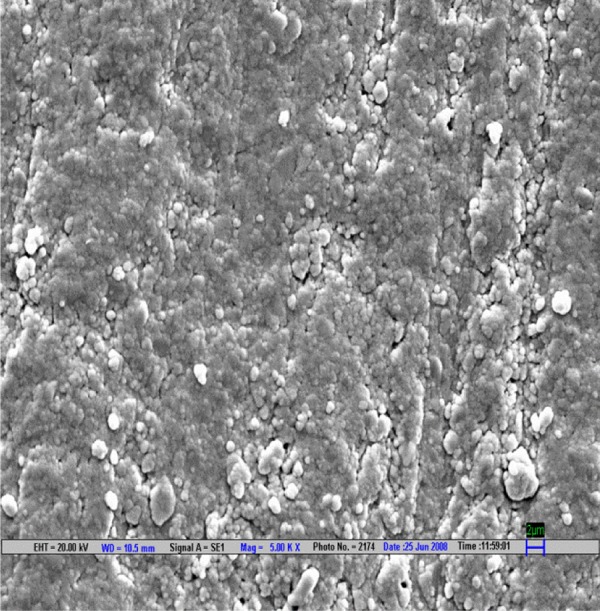
Scanning electron microscopic image of EX composite polished by Enhance polishing system (magnification 5000*)

Stoddard and Johnson^14^ suggested that because of the variation in filler particles and types of resin, it is important to pair a resin composite with a matching polishing system. In this study, we are only evaluating surface roughness which is one of the several parameters that influence the clinical surface quality of a restoration. Additional factors affecting the polishing results may include the amount of pressure utilized while polishing, the orientation of the abrading surface and the amount of time spent both with each abrading surface and abrasive material should be considered for evaluating the clinical efficiency among the polishing systems available today.

**Fig. 9:**
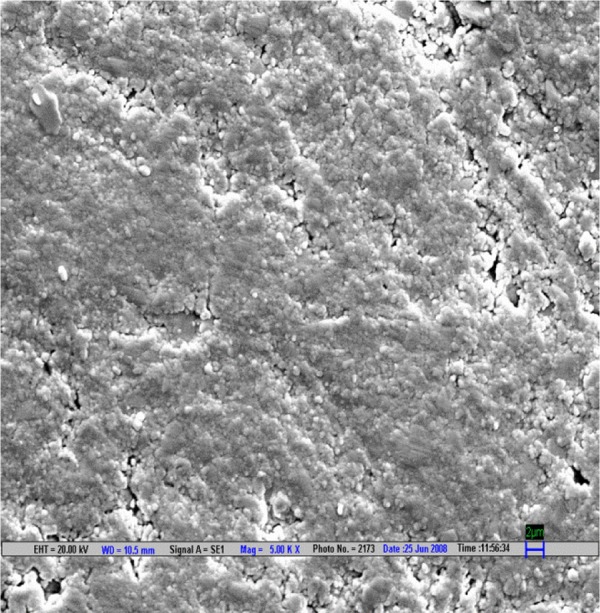
Scanning electron microscopic image of Filtek Z 250 composite polished by Enhance polishing system (magnification 5000*)

## CONCLUSION

- Group I (Shofu polishing system) shows superior polishability than the groups II (Sof-Lex) and III (Enhance).
- Group III (Enhance polishing system) showed the least polishability among all the groups.
- Nanofilled composite (CX) showed better polishability than the microfilled and hybrid composites.
- The hybrid (Filtek Z-250) composite showed the least polishability than the nanofilled and microfilled composites.
